# Pars Plana Ahmed Valve Implantation for Vitrectomized Eyes With Refractory Glaucoma

**DOI:** 10.3389/fmed.2022.883435

**Published:** 2022-04-25

**Authors:** Xiaoli Xiang, Pan Xiao, Jingjing Yu, Yihong Cao, Tingwang Jiang, Zhengru Huang

**Affiliations:** ^1^Department of Ophthalmology, The Affiliated Changshu Hospital of Xuzhou Medical University, Changshu, China; ^2^Department of Key Laboratory, The Affiliated Changshu Hospital of Xuzhou Medical University, Changshu, China; ^3^Gusu School, Nanjing Medical University, Suzhou, China

**Keywords:** Ahmed valve implantation, refractory glaucoma, surgery, ophthalmology, vitrectomy

## Abstract

This study aimed to analyze the surgical outcomes of pars plana Ahmed valve implantation in vitrectomized eyes with refractory glaucoma. We performed a retrospective case review of consecutive patients with refractory glaucoma after undergoing pars plana vitrectomy who underwent pars plana Ahmed valve implantation between July 2019 and December 2020 at the glaucoma unit of the Affiliated Changshu Hospital of Xuzhou Medical University (Changshu, China). All the patients were followed up for ≥12 months postoperatively. We recorded pre- to postoperative changes in best-corrected visual acuity (BCVA), intraocular pressure (IOP), number of anti-glaucoma medication, corneal endothelial count, and surgical complications, if any. There was a significant improvement in the median BCVA from 2.30 (0.87, 2.30) logMAR preoperatively to 1.70 (0.70, 2.30) logMAR at discharge and 1.0 (0.52, 1.85) at final examination (*p* = 0.011, *p* = 0.001). Compared with the preoperative IOP level, there was a significant decrease in the postoperative IOP at each postoperative time point (*p* < 0.001). There was a significant reduction in the median number of anti-glaucoma drugs (including postoperative ocular massage), from 3.00 (2.00, 3.00) preoperatively to 0.00 (0.00, 1.00) at the last follow-up postoperative examination (*p* < 0.001). A 29-year-old woman with proliferative diabetic retinopathy who underwent surgical treatment at 5 months postoperatively for fibrous wrapping formed around the plate of the Ahmed valve showed an IOP of 14 mmHg at the last follow-up. Our findings indicated that pars plana Ahmed valve implantation can be safely performed for managing vitrectomized eyes with refractory glaucoma.

## Introduction

Refractory glaucoma, including neovascular glaucoma (NVG), aphakic or intraocular lens eye glaucoma, failed filtering surgery-induced glaucoma, congenital juvenile glaucoma, glaucoma combined with uveitis, traumatic glaucoma, and glaucoma after vitrectomy, remains an issue in the field of glaucoma treatment (1). The glaucoma drainage implant (GDI) is an external filtering anti-glaucoma surgical device that is especially suitable for refractory glaucoma (2). However, implanting this device may involve several complications (3). GDIs can be implanted in the anterior chamber, ciliary sulcus, or vitreous cavity. In some cases of corneal disease, iris abnormalities, or peripheral anterior synechia, the tube cannot be placed in the anterior chamber (4); tube implantation into the vitreous cavity is a viable option for resolving refractory tube-related anterior complications (5).

The Ahmed glaucoma valve (AGV) was introduced in 1993 as the first GDI with a one-way valve mechanism that allows immediate postoperative flow and prevents ocular hypotension (6). Therefore, an AGV may be preferred for patients at risk of low intraocular pressure (IOP), including those who have undergone vitrectomy, and may be effective for treating vitrectomized eyes with refractory glaucoma (7). The AGV implantation technique takes advantage of the relatively wide and easily separated posterior superior scleral space. Specifically, a large AGV drainage tray is implanted into the posterior superior scleral space (mostly between the external rectus and superior rectus sheath) to reduce fascial interference and form a permanent functional filtering area around the drainage tray to allow sufficient IOP control (8).

Moreover, AGV implantation is the only restricted GDI approved by the National Medical Products Administration for listing in China (8). This study aimed to analyze the surgical outcomes of pars plana Ahmed valve implantation in vitrectomized eyes with refractory glaucoma in patients from the Changshu city of Jiangsu province, China.

## Materials and Methods

This study was approved by the Institutional Review Board of Changshu Hospital of Xuzhou Medical University (Changshu, China; approval number: 2016034). It was conducted in accordance with the Declaration of Helsinki. Informed consent for surgery and study participation obtained from all the patients before treatment was part of their medical records.

### Patients

This retrospective observational case series included consecutive patients with refractory glaucoma after pars plana vitrectomy (PPV) who underwent pars plana Ahmed valve implantation between July 2019 and December 2020 at the glaucoma unit of the Affiliated Changshu Hospital of Xuzhou Medical University (Changshu, China). The inclusion criteria were as follows: (1) a diagnosis of glaucoma, with IOP not controlled by confirmed medical treatment (IOP ≥ 30 mmHg despite maximal tolerable medication use); (2) having undergone total vitrectomy, with the vitreous cavity being filled with water or gas; and (3) the AGV tube was inserted into the vitreous cavity through the pars plana. Bilateral eye surgery was performed, in which case we recorded the eye in which the AGV was initially implanted. The exclusion criteria were loss to follow-up or a follow-up period <12 months.

### Assessments

We obtained ophthalmic examination results from inpatient and outpatient medical records and additionally recorded the history of glaucoma.

All the patients underwent the following preoperative procedures: (1) slit-lamp microscopy; (2) fundus examination; (3) B ultrasound (Quantel Medical Aviso); (4) ultrasonic biological microscopy (Aviso, Quantel Medical, France); (5) Humphrey field of vision assessment (Carl Zeiss, Germany); (6) retinal nerve fiber layer thickness (Cirrus HD-OCT5000, Carl Zeiss, Germany); and (7) corneal endothelioscopy (SP-3000P, Topcon, Japan).

Additionally, we recorded (1) the best-corrected visual acuity (BCVA); (2) IOP; (3) corneal endothelial count; (4) intraoperative complications; (5) postoperative complications; (6) time of occurrence; and (7) the type and quantity of anti-glaucoma medication used pre- and postoperatively. Furthermore, eye massage was included as an anti-glaucoma medication.

Best-corrected visual acuity was recorded in decimal form and converted to logMAR for statistical analysis (logMAR = log [1/VA]) (9). IOP was measured using Goldmann applanation tonometry. All patients with increased IOP after AGV implantation underwent treatment, including eye massage and anti-glaucoma medication, and anti-glaucoma surgery would be performed as required.

### Surgical Procedures

**FIGURE 1:**
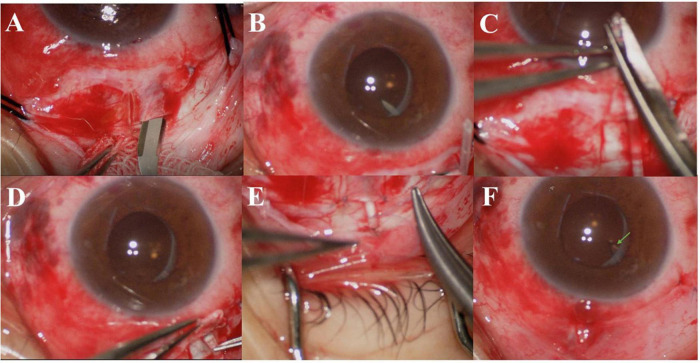
**(A)** A rectangular, limbal-based half-thickness scleral flap was created. **(B)** The vitreous cavity was accessed using a 22-gauge needle at 3.5–4 mm behind the corneal limbus under the scleral flap. **(C)** The tube length was trimmed. **(D)** The tube was placed into the vitreous cavity at 3–4 mm through the needle track, and the tube inside the vitreous chamber can be seen under the dilated pupil. **(E)** The drainage tube after the scleral tunnel was fixed on the scleral surface using 8–0 Vicryl sutures. **(F)** The conjunctiva was sutured using 8–0 Vicryl sutures, and the tube (arrow) inside the vitreous chamber can be seen under the dilated pupil.

All surgeries were performed by an experienced ophthalmologist (ZH) using AGV Model FP7 (New World Medical Inc., Rancho Cucamonga, CA, United States). All patients underwent surgery under peribulbar anesthesia. The conjunctival flap was made based on the conjunctival fornix. We created a rectangular limbal-based half-thickness scleral flap. After the AGV was primed, it was placed 10 mm behind the corneal limbus in the superotemporal quadrant between the superior and lateral rectus muscles. The AGV plate was secured to the sclera using a 5–0 braided polyester suture. The vitreous cavity was accessed with a 22-gauge needle at 3.5–4 mm behind the corneal limbus under the scleral flap. The tube length was trimmed, and the tube was placed in the vitreous cavity 3–4 mm through the needle track. The tube bevel was directed toward the vitreous cavity. The tube and its entry site were covered using an autologous scleral flap. The scleral flap was reset, and both ends were sutured using 8–0 Vicryl sutures without tension. The drainage tube after the scleral tunnel was fixed on the scleral surface using 8–0 Vicryl sutures. The conjunctiva was sutured using 8–0 Vicryl sutures (Figure 1).

### Statistical Analysis

Data are expressed as mean ± standard deviation (SD), median (P25, P75), or number (percentage;%). For quantitative variables, we used histograms, box plots, and Shapiro–Wilk tests to evaluate the distribution normality. Changes in variables between time points were analyzed using paired sample *t*-test for normally distributed variables or the related samples Wilcoxon signed-rank test and Friedman’s two-way analysis of variance for non-normally distributed variables. The relationship between categorical variables was analyzed using the chi-squared test. We performed a Kaplan–Meier survival analysis of the operation success rate (criteria for success: 8 mm Hg ≤ postoperative IOP ≤ 21 mm Hg, without drug treatment). Log-rank test was used to compare the differences among groups in the distribution of survival time. All statistical analyses were performed using SPSS (version 19.0; SPSS Inc., Chicago, IL, United States) and GraphPad Prism 8.0 (GraphPad Software Inc., San Diego, CA, United States). Statistical analyses were performed with a 5% significance level; moreover, statistical significance was set at a two-sided *p*-value < 0.05.

## Results

### Baseline Characteristics

We included 21 eyes from 21 patients with vitrectomized refractory glaucoma. Among them, there were 11 cases (11 eyes) of NVG. The mean age was 61.95 ± 16.42 years, and 11 (52.4%) patients were female. There were 10 right eyes. Furthermore, there were 6.98 ± 11.20 months from the last surgery until the pars plana Ahmed valve implantation surgery (Table 1).

**TABLE 1:** Baseline characteristics of patients before pars plana Ahmed valve implantation surgery.

Parameters	Total	NVG	Non-NVG	*p*-value
Number of eyes	21	11	10	
Right-side N (%)	10(47.6)	5(45.5)	5(50)	
Female, eyes N (%)	11(52.4)	7(63.6)	4(40)	
Age (years) Mean ± SD	61.95 ± 16.42	56.55 ± 20.16	67.90 ± 8.53	0.168
**Glaucoma type N (%)**				
Neovascular (in the angle-closure stage)	11(52.4)	11(100)		
Open angle	3(14.3)		3(30)	
Traumatic	3(14.3)		3(30)	
Malignant	2(9.5)		2(20)	
Steroid response	2(9.5)		2(20)	
**Indication of vitrectomy N (%)**				
RVO	4(19)	4(36.4)		
PDR	7(33.3)	7(63.6)		
Macular disease	5(23.8)		5(50)	
Trauma	3(14.3)		3(30)	
Malignant glaucoma	2(9.5)		2(20)	
Interval between PPV and AGV (months) Mean ± SD	6.98 ± 11.20	4.10 ± 3.84	10.15 ± 15.54	0.437
Follow-up period (months) Mean ± SD	17.67 ± 5.33	18.83 ± 5.64	16.50 ± 4.99	0.321
Pre-AGV IOP (mmHg) Mean ± SD	44.76 ± 12.63	44.64 ± 12.53	44.90 ± 13.43	0.858
Pre-AGV BCVA (logMAR) Mean ± SD	1.75 ± 0.79	2.15 ± 0.56	1.31 ± 0.78	0.007
Pre-AGV number of medication Mean ± SD	2.71 ± 0.46	2.73 ± 0.47	2.70 ± 0.48	0.893

*NVG, neovascular glaucoma; AGV, Ahmed glaucoma valve; BCVA, best-corrected visual acuity; IOP, intraocular pressure; PPV, pars plana vitrectomy; SD, standard deviation; RVO, retinal vein occlusion; PDR, proliferative diabetic retinopathy.*

### Best-Corrected Visual Acuity

There was a significant improvement in the BCVA from a median of 2.30 (0.87, 2.30) logMAR preoperatively to 1.70 (0.70, 2.30) logMAR at discharge and 1.0 (0.52, 1.85) at the final examination (*p* = 0.011 and *p* = 0.001, respectively, Table 2). Overall, there was a correlation between BCVA at last follow-up and at baseline (Spearman correlation; *r* = 0.729, *p* < 0.001). Preoperative BCVA, BCVA at discharge, and BCVA at last follow-up of patients without NVG were better than those of patients with NVG, and the differences were statistically significant (*p* = 0.007, *p* = 0.005, and *p* = 0.018, respectively, Table 3). However, stratum analysis showed no correlation between visual acuity at last follow-up and at baseline in patients with NVG (*r* = 0.235, *p* = 0.487); furthermore, there was a correlation between visual acuity at last follow-up and at baseline in patients without NVG (*r* = 0.708, *p* = 0.022).

**TABLE 2:** Intraocular pressure (IOP), best-corrected visual acuity (BCVA), and number of anti-glaucoma drugs at different time points for patients who underwent pars plana Ahmed valve implantation surgery.

	Mean ± SD	Median (P25, P75)	Median difference (95% CI)	*P*-value
**IOP (mmHg) (*n* = 21)**				
Pre-AGV	44.76 ± 12.64	45.00 (32.00, 60.00)		
1 day	16.81 ± 5.97	15.00 (13.50, 17.50)	28.50 (22.00, 34.00)	<0.001
1 week	17.10 ± 6.39	15.00 (13.00, 20.00)	29.00 (23.00, 34.50)	<0.001
1 month	15.81 ± 4.30	15.00 (13.50, 18.00)	29.50 (23.00, 35.00)	<0.001
3 months	17.09 ± 4.48	16.00 (14.00, 20.00)	28.00 (22.00, 34.50)	<0.001
6 months	16.38 ± 3.97	16.00 (13.00, 19.00)	28.00 (22.50, 34.50)	<0.001
Last follow-up	17.06 ± 3.43	16.00 (14.00, 19.00)	27.50 (22.50, 33.50)	<0.001
**BCVA (logMAR) (*n* = 21)**				
Pre-AGV	1.75 ± 0.79	2.30 (0.87, 2.30)		
At discharge	1.55 ± 0.82	1.70 (0.70, 2.30)	0.11(0.00, 0.30)	0.011
Last follow-up	1.29 ± 0.80	1.00 (0.52, 1.85)	0.41 (0.15, 0.73)	0.001
**Number of anti-glaucoma drugs (*n* = 21)**				
Pre-AGV	2.71 ± 0.46	3.00 (2.00, 3.00)		
Last follow-up	0.48 ± 0.60	0.00 (0.00, 1.00)	2.50 (2.00, 2.50)	<0.001
**Corneal endothelial count (cells/mm^2^) (*n* = 19)**				
1 week	1668.35 ± 376.32	1760.70 (1394.00,1885.60)		
6 months	1632.75 ± 369.67	1691.50 (1379.80,1895.20)	24.48 (0.15, 65.85)	0.053

*AGV, Ahmed glaucoma valve; BCVA, best-corrected visual acuity; IOP, intraocular pressure; SD, standard deviation; CI, confidence interval.*

**TABLE 3:** Comparison of IOP, BCVA, and number of anti-glaucoma drugs at different time points between NVG and non-NVG patients.

	NVG (*n* = 11)	Non-NVG (*n* = 10)	*P*-value
**IOP (mmHg) Mean ± SD/Median (P25, P75)**			
Pre-AGV	44.64 ± 12.53/45.00 (34.00, 60.00)	44.90 ± 13.43/47.50 (29.50, 60.00)	0.858
1 day	18.45 ± 7.81/16.00 (14.00, 22.00)	15.00 ± 2.11/15.00 (13.00, 17.25)	0.337
1 week	15.36 ± 4.39/13.00 (12.00, 20.00)	19.00 ± 7.85/15.50 (14.00, 21.25)	0.204
1 month	15.27 ± 4.03/16.00 (10.00, 20.00)	16.40 ± 4.72/15.00 (13.75, 17.25)	0.972
3 months	16.55 ± 3.39/16.00 (14.00, 20.00)	17.70 ± 5.58/16.00 (14.25, 24.25)	0.723
6 months	16.73 ± 3.44/17.00 (13.00, 19.00)	16.00 ± 4.64/15.00 (12.75, 17.50)	0.458
Last follow-up	17.64 ± 3.35/19.00 (14.00, 20.00)	16.50 ± 3.60/14.00 (15.00, 19.00)	0.374
**BCVA (logMAR) Mean ± SD/Median (P25, P75)**			
Pre-AGV	2.15 ± 0.56/2.30 (1.85, 2.60)	1.31 ± 0.78/0.87 (1.85, 2.30)	0.007
At discharge	2.01 ± 0.59/2.30 (1.70, 2.60)	1.04 ± 0.76/0.70 (0.52, 1.85)	0.005
Last follow-up	1.66 ± 0.65/1.70 (1.00, 2.30)	0.87 ± 0.77/0.52 (0.30, 1.74)	0.018
**Number of anti-glaucoma drugs Mean ± SD/Median (P25, P75)**			
Pre-AGV	2.73 ± 0.47/3.00 (2.00, 3.00)	2.70 ± 0.48/3.00 (2.00, 3.00)	0.893
Last follow-up	0.36 ± 0.51/0.00 (0.00, 1.00)	0.60 ± 0.70/0.50 (0.00, 1.00)	0.443
**Corneal endothelial count (cells/mm^2^) Mean ± SD/Median (P25, P75)**			
1 week	1694.97 ± 360.89/1760.70 (1394.00, 1926.40)	1631.74 ± 418.87/1692.15 (1284.85, 1872.95)	0.680
6 months	1656.34 ± 366.65/1691.50 (1379.80, 1949.60)	1600.34 ± 396.58/1696.35 (1239.25, 1886.40)	0.717

*NVG, neovascular glaucoma; AGV, Ahmed glaucoma valve; BCVA, best-corrected visual acuity; IOP, intraocular pressure; SD, standard deviation.*

### Intraocular Pressure

**FIGURE 2:**
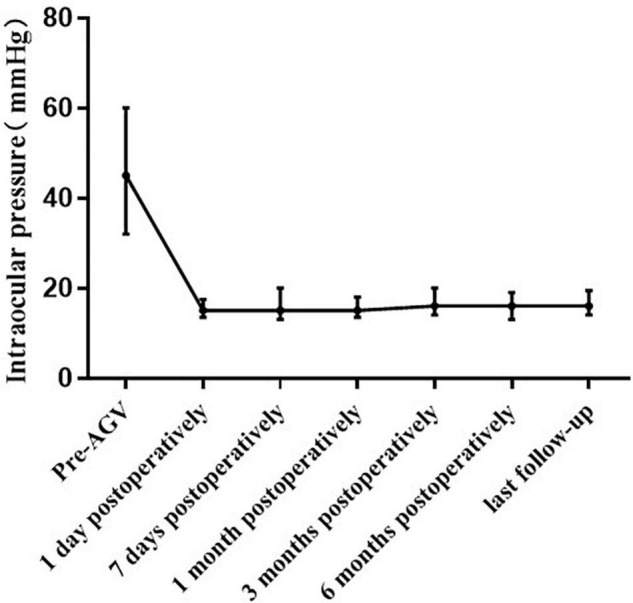
Line chart of the intraocular pressure before and after pars plana Ahmed valve implantation, with postoperative assessments at 1 day, 7 days, 1 month, 3 months, 6 months, and last follow up.

The median IOP was 45.00 (32.00, 60.00) mmHg preoperatively and 15.00 (13.50, 17.50) mmHg at 1 day postoperatively. The IOP at 1 day postoperatively was lower than the preoperative IOP in all eyes. The IOP was 15.00 (13.00, 20.00) mmHg at 7 days postoperatively. The IOP at 7 days postoperatively was higher than the preoperative IOP in one eye. The median IOP at 1 month postoperatively was 15.00 (13.50, 18.00) mmHg, which was higher than the preoperative IOP in one eye (aforementioned patient). The IOP was 16.00 (14.00, 20.00) mmHg at 3 months postoperatively, 16.00 (13.00, 19.00) mmHg at 6 months postoperatively, and 16.00 (14.00, 19.00) mmHg at the final examination. Compared with the preoperative IOP, the postoperative IOP significantly decreased at each postoperative time point (*p* < 0.001; Figure 2 and Table 2). However, there were no significant differences in the IOP values among the postoperative time points (*p* = 0.616). There was no significant difference in the IOP between patients with and without NVG at each time point (Table 3).

**FIGURE 3:**
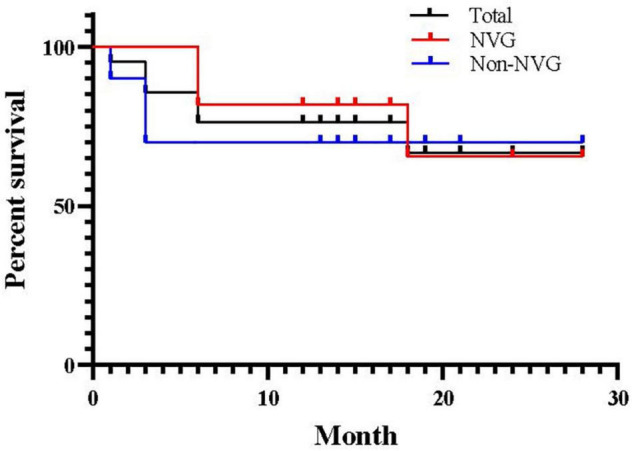
Kaplan–Meier survival curve for success after pars plana Ahmed valve implantation. Success was defined as 8 mmHg ≤ postoperative intraocular pressure ≤ 21 mmHg without medication.

Figure 3 shows the Kaplan–Meier survival curve. The total success rates were 85.71% at 3 months, 76.19% at 6 months, and 71.43% at 1 year postoperatively. There were no significant differences in the survival time distributions between patients with and without NVG (*p* = 0.691).

### Number of Anti-glaucoma Drugs

At 1 year postoperatively, the IOP was maintained below 20 and 15 mmHg in 18 and 10 patients, respectively, without any anti-glaucoma drugs. There was a significant reduction in the use of anti-glaucoma drugs (including postoperative ocular massage) from a median of 3.00 (2.00, 3.00) preoperatively to 0.00 (0.00, 1.00) at the last follow-up (*p* < 0.001, Table 2). There were no statistically significant differences in the number of anti-glaucoma drugs used preoperatively and at the last follow-up between patients with and without NVG (*p* = 0.893 and *p* = 0.443, respectively, Table 3).

### Corneal Endothelial Count

Corneal endothelial counts could not be preoperatively measured in two patients with trauma, two patients with malignant glaucoma, and 11 patients with NVG. Therefore, we measured the corneal endothelium count in all patients at 1 week postoperatively, but the results were still unavailable in two patients with trauma. There was no significant difference in the corneal endothelial count at 6 months postoperatively and 1 week postoperatively (*p* = 0.334, Table 2). There were no statistically significant differences in the corneal endothelial count at 6 months postoperatively and 1 week postoperatively between patients with and without NVG (*p* = 0.680 and *p* = 0.717, respectively, Table 3).

### Complications

A 29-year-old woman with proliferative diabetic retinopathy underwent surgical treatment at 5 months postoperatively for fibrous wrapping formed around the plate of the AGV, with the IOP at the last follow-up being 14 mmHg. Two patients with trauma underwent intraocular lens suspension at 6 months after Ahmed valve implantation, with stable postoperative ocular condition. Patients with retinal vein occlusion and proliferative diabetic retinopathy continued to receive postoperative anti-vesicular endothelial growth factor injections, as required. None of the patients showed other complications, including corneal endothelial decompensation, drainage tube shift, drainage tube exposure, drainage tube blockage, anterior chamber inflammation, or infectious endophthalmitis, during the follow-up period.

## Discussion

This retrospective case review assessed the pars plana Ahmed valve implantation in patients with refractory glaucoma post-vitrectomy from the Changshu city of Jiangsu province, China. Our findings indicated a significant improvement in IOP and medication consumption postoperatively. The BCVA of patients without NVG was better than that of patients with NVG at each time point. Our results suggest that pars plana Ahmed valve implantation is an effective treatment for vitrectomized eyes with refractory glaucoma, including NVG.

There has been considerable progress in PPV for treating posterior segment ocular disease (10). However, glaucoma remains a common complication after vitreoretinal surgery for various reasons. PPV is associated with an increased risk of open-angle glaucoma, and there is a risk of increased IOP after vitreoretinal surgery (11–15). Possible causes include increased oxidative stress in the vitreous cavity caused by post-vitrectomy trabecular injury, trabecular scarring caused by minor injuries during vitrectomy, progression of neovascularization, and the use of silicone oil in vitreoretinal surgery (13, 16, 17). However, the relationship between PPV and open-angle glaucoma remains unclear (18, 19).

Postoperative hypotony is a common complication of AGV implantation in vitrectomized eyes. Possible related factors include aqueous leakage from the drainage tube periphery and valve device destruction upon glaucoma valve implantation. However, after the formation of filtering blebs, most eyes return to normal (6, 8). The absence of early stage postoperative hypotony may be associated with the use of a 22-gauge needle to access the vitreous cavity at 3.5-4 mm behind the corneal limbus under the scleral flap and the use of 8–0 Vicryl sutures to intraoperatively fix the drainage tube on the sclera so that postoperative hypotony caused by leakage from the puncture tunnel into which the tube was inserted was unlikely to occur.

Choroidal detachment is also a common postoperative complication, mainly due to perioperative IOP fluctuations. It is more common in patients with persistent low IOP caused by preoperative hypertension and sudden drop of hypotension, excessive drainage, or puncture tunnel leakage intraoperatively (8). Therefore, intra- and postoperative IOP stabilization need to be emphasized. There was no postoperative hypotony in our cases. As a result, no complications, such as choroidal detachment, occurred postoperatively.

In our study, patients showed mild postoperative anterior chamber inflammatory response, with a stable anterior chamber and no postoperative hypotony and choroidal leakage or detachment. Complete peripheral vitrectomy is essential for the continued success of the AVG implantation procedure to avoid vitreous tube obstruction, which causes failure of IOP control or tearing due to the application of traction to the retina.

A previous study reported that the mean surgical interval was 1.8 ± 2.3 months and 12.41 ± 16.2 months in the NVG and non-NVG groups, respectively (6). NVG was found to occur 151 days after PPV, with 56% of the patients requiring surgery (16). Another study reported that the mean interval between PPV and AGV implantation was 7.5 ± 2.2 months (20). Our patients included both NVG and others; moreover, the overall mean interval between PPV and AGV implantations was 6.98 ± 11.20 (0.1–52) months, with a mean interval between PPV and AGV implantations of 4.10 ± 3.84 (0.10–12) months in the NVG group. In the non-NVG group, the mean interval between PPV and AGV implantations was 10.15 ± 15.54 (0.25–52) months.

Excessive fibrous wrapping of tenon’s capsule around the drainage plate is the main reason for surgery failure, which affects filtration function (21). Patients with a high IOP during the early postoperative period were treated using ocular massage, which not only reduces IOP but also facilitates soaking of the fascia tissue by drained aqueous humor to prevent its proliferation. In the early postoperative period, ocular massage is an important measure for effectively preventing the blockage of the drainage tube head. After the function of the AGV is stabilized, intermittent ocular massage can prevent severe obstruction of the lumen caused by the accumulation of metabolic substances in the eye (8). Ocular massage was performed to avoid the drainage implantation site and cornea, as well as to avoid drainage tube displacement caused by direct compression (7). Since the drainage site in our patients was located in the superior temporal quadrant, we recommended that they look above and massage below. In our study, a young female patient with NVG developed a fibrous cyst formed around the plate of the AGV. We excised the outer cyst wall and strengthened postoperative eyeball massage, and the patient’s IOP remained stable.

Corneal endothelial decompensation is also a serious complication of AGV implantations. The drainage tube in the anterior chamber is either in contact or proximal with the corneal endothelium; alternatively, it makes contact with the corneal endothelium when postoperatively massaging the eyeball. This causes corneal endothelial damage and functional decompensation. Specifically, the corneal endothelium of patients with a long-term shallow anterior chamber and high IOP is often in poor condition; additionally, these patients are prone to further postoperative damage. The drainage tube was implanted into the vitreous cavity from the pars plana to avoid touching and damaging the cornea. Moreover, stable fixation of the drainage tube is ensured to reduce its displacement when the eyeball is rotated or massaged, which can effectively prevent complications. In our study, the drainage tube was implanted and firmly fixed into the vitreous cavity to avoid possible contact between the drainage tube and cornea, as well as to reduce disturbance to the anterior chamber caused by foreign bodies and surgery. Additionally, there was no significant difference in the corneal endothelial count preoperatively and at 6 months postoperatively.

In contrast to the thin-walled bleb after trabeculectomy, the bleb over the plate is far from the limbus. Furthermore, it has a thick and fibrous capsule, which could reduce the chances of bleb leaks or bleb-related infections (22). Exposure of drainage tubes can cause severe complications, including low IOP and endophthalmitis, which usually requires surgical repair (23). Pars plana tube implantation avoids the thin limbal region of the conjunctiva and reduces the extraocular tube length, which prevents complications (5). None of our patients showed drainage tube exposure. Furthermore, the tube could only be seen during dilation examination; therefore, it did not affect the patient’s appearance.

Additionally, visual acuity is not considered in the success criteria of AGV implantation, since the treatment goal for refractory glaucoma is normalizing the IOP (24, 25). Visual acuity after glaucoma surgery varies according to glaucoma severity, surgical complications, and other factors. During the follow-up period, some of our patients continued anti-vesicular endothelial growth factor therapy and intraocular lens implantation, improving vision at the last follow-up compared with before surgery.

## Conclusion

The present findings demonstrated the short-term efficacy of pars plana Ahmed valve implantation in vitrectomized eyes with refractory glaucoma. We observed that pars plana Ahmed valve implantation can be safely performed for managing vitrectomized eyes with refractory glaucoma, with low surgical requirements, large allowable space, and few complications. However, further studies are warranted to evaluate the long-term efficacy of the procedure.

Although all our patients showed post-vitrectomy refractory glaucoma, their conditions were heterogenous. Further, this was a retrospective study with a small sample size, no controls, and a short follow-up period. Therefore, there is a need for further studies, preferably randomized controlled trials, with large sample sizes and longer follow-up periods.

## Data Availability Statement

The raw data supporting the conclusions of this article will be made available by the authors, without undue reservation.

## Ethics Statement

The studies involving human participants were reviewed and approved by the Institutional Review Board of Changshu Hospital of Xuzhou Medical University (Changshu, China; approval number: 2016034). The patients/participants provided their written informed consent to participate in this study.

## Author Contributions

XX, PX, JY, and ZH: conception and design. XX, PX, JY, and YC: data collection and collation. XX and TJ: data analysis and interpretation. XX: manuscript writing. XX and ZH: data interpretation and final review of the manuscript. All authors revised and approved the submitted manuscript.

## Conflict of Interest

The authors declare that the research was conducted in the absence of any commercial or financial relationships that could be construed as a potential conflict of interest.

## Publisher’s Note

All claims expressed in this article are solely those of the authors and do not necessarily represent those of their affiliated organizations, or those of the publisher, the editors and the reviewers. Any product that may be evaluated in this article, or claim that may be made by its manufacturer, is not guaranteed or endorsed by the publisher.

## Abbreviations

AGV: Ahmed glaucoma valve
BCVA: best-corrected visual acuity
GDI: glaucoma drainage implant
IOP: intraocular pressure
NVG: neovascular glaucoma
PPV: pars plana vitrectomy
SD: standard deviation.
